# Macular Thickness Variability in Primary Open Angle Glaucoma Patients using Optical Coherence Tomography

**DOI:** 10.5005/jp-journals-10008-1154

**Published:** 2014-01-16

**Authors:** Anjali Sharma, Prakashchand Agarwal, P Sathyan, VK Saini

**Affiliations:** Assistant Professor, Department of Ophthalmology, People's College of Medical Sciences and Research Centre, Bhopal, Madhya Pradesh, India; Assistant Professor, Department of Ophthalmology, People's College of Medical Sciences and Research Centre, Bhopal, Madhya Pradesh, India; Professor and Head, Department of Ophthalmology, Sathyan Eye Care Hospital and Coimbatore Glaucoma Foundation, Coimbatore, Tamil Nadu, India; Professor and Head, Department of Ophthalmology, People's College of Medical Sciences and Research Centre, Bhopal, Madhya Pradesh, India

**Keywords:** Macular thickness, Glaucoma, Optical coherence tomography.

## Abstract

**Aim:** To compare the difference of retinal macular thickness and macular volume using optical coherence tomography (OCT) in primary open angle glaucoma (POAG) patients with the normal subjects.

**Materials and methods:** This observational case control study included primary open angle glaucoma (POAG) patients (n = 124 eyes) and healthy subjects in the control group (n = 124 eyes). All subjects underwent detailed history, general and systemic exami -nation. Complete ocular examination included best corrected visual acuity (BCVA), slit lamp examination, intraocular pressure (IOP), central corneal thickness, gonioscopy, dilated fundus biomicroscopy. Field analysis was done by white on white Humphrey Field Analyzer (Carl Zeiss).

Optical coherence tomography imaging of macular area was performed using Stratus OCT (OCT 3, Version 4, Carl Zeiss Inc, Dublin, California, USA). In both these groups, parameters analyzed were macular thickness, inner macular thicknesses (IMT), outer macular thicknesses (OMT), central macular thick ness (CMT) and total macular volume (TMV).

**Results:** The POAG group had significantly decreased values of TMV, OMT and IMT, compared to control group, while there was no difference in CMT, presumably due to absence of ganglion cells in the central part. Thus, macular thickness and volume parameters may be used for making the diagnosis of glaucoma especially in patients with abnormalities of disc.

**Conclusion:** Macular thickness parameters correlated well with the diagnosis of glaucoma.

**How to cite this article:** Sharma A, Agarwal P, Sathyan P, Saini VK. Macular Thickness Variability in Primary Open Angle Glaucoma Patients using Optical Coherence Tomography. J Current Glau Prac 2014;8(1):10-14.

## INTRODUCTION

Glaucoma is a progressive optic neuropathy characterized by a loss of retinal ganglion cells (RGC)^1^ which results in characteristic visual field impairment.^2^ Glaucoma is diagnosed clinically by observing optic disk changes and by measurement of visual function with perimetry. Perimetry changes appear when up to 70% or more retinal nerve fiber layer (RNFL) is damaged so to detect preperimetric glaucoma studies are focused now to evaluate RNFL and ganglion cells to detect glaucoma early.^3^

The macula contains over 50% of all retinal ganglion cells and is an ideal area for detection of early cell loss and changes over the time because of high cell density.^4,5^ In the macular area, ganglion cells are arranged in 4 to 6 layers making up 30 to 35% of retinal macular thickness, so that the loss of macular ganglion cells results in significant retinal or retinal nerve fiber layer thinning.^6-8^ Several studies indicated that in glaucomatous eyes decrease in macular thickness and volume are due to loss of RGCs and that this finding correlate with RNFL thickness and visual field defects.^9,10^ Recent studies imply that thinning of RNFL is related to the thinning of macular ganglion cell complex (GCC), which is defined as three innermost retinal layers: (1) RNFL (made of ganglion cell axons), (2) ganglion cell layer (GCL) made of ganglion cell bodies and (3) the inner plexiform layer (IPL) made out of ganglion cell dendrites. All three layers of ganglion cell complex are significantly thinner in glaucoma patients, refecting the proportion of dead ganglion cells,^11^ although Tan et al found that residual glial tissue maintains 50% thickness even when all ganglion cells are lost.^12^

In our study, we have evaluated inner macular thickness (IMT) (Central 3 mm), outer macular thickness (OMT) (outer 6 mm zone) and total macular volume (TMV) in primary open angle glaucoma (POAG) patients and compared it with healthy subject in a case control observational method.

## MATERIALS AND METHODS

A total of 144 subjects were recruited for the study. Group A included 76 patients of primary open angle glaucoma (POAG, n = 124 eyes) and group B included 68 normal subjects (Controls, n = 124 eyes). The study was conducted at glaucoma clinic of Aravind Eye Hospital, Coimbatore, Tamil Nadu. Written Informed consent was obtained from each participant before enrolment.

Exclusion criteria included diabetic retinopathy, macular degeneration, macular edema, epiretinal membrane, retinal detachment, cataract, high myopia (greater than 4.00 D Sph. or 2.00 D Cyl), presence of nonglaucomatous optic nerve diseases and previous ocular surgery or trauma. We have also excluded all patients with secondary glaucoma angle closure glaucoma or operated cases of POAG.

The diagnosis of POAG was based on glaucomatous damage to the optic disk (optic nerve head cupping) and abnormal visual fields and IOP values. All eyes with glaucoma had visual field loss (mean deviation of glaucomatous eyes on visual field testing was –6.01 dB to –13.73 dB, according to Hodapp-Parrish-Anderson Grading scale of severity of visual field defect) in at least two consecutive examinations tested by automated perimetry.^13^

The control group included the subjects with no history of glaucoma or retinal pathology IOP of < 21 mm Hg, normal optic nerve head appearance and normal visual field testing results (mean defect –2.0 to +2.0 dB) normal eyes served as control group.

All subjects underwent detailed history, general and systemic examination. Complete ocular examination included best corrected visual acuity (BCVA), slit-lamp exami nation, intraocular pressure (IOP) measurement using Goldmann Applanation Tonometery (corrected according to central corneal thickness), central corneal thickness was done with ultrasound pachymeter (PAC Scan 300P digital biometric ruler, Sonomed), gonioscopy, dilated fundus biomicroscopy using +90 diopter lens (dilated with tropicamide 0.5%). Field analysis was done by white on white Humphrey Field Analyzer (Carl Zeiss, USA).

All patients were scanned with Stratus OCT (Stratus OCT Model 300, Carl Zeiss, Meditec Inc. Dublin CA, USA). We analyzed the changes of macular thickness para meters and macular volume.

**Fig. 1:**
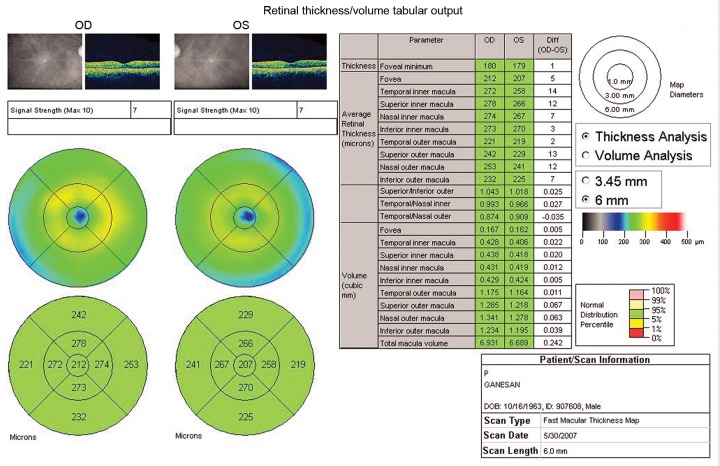
Macular thickness map

*OCT analysis*: Fast macular thickness scan was obtained for all the subjects using OCT (Fig. 1). The data thus obtained was analyzed using independent ‘t' test for statistical significance. A p-value less than 0.05 was considered statistically significant.

## RESULTS

We have investigated 144 subjects. Group A included 76 patients of primary open angle glaucoma (POAG, n = 124 eyes) and group B included 68 normal subjects (controls, n = 124 eyes). Demographic data is expressed in Table 1.

### Inner Macular Thickness

The mean inner macular thickness (central 3 mm) of the superior, inferior, temporal and nasal quadrants among groups A and B are expressed in the Table 2. There is statistically significant difference (p < 0.05) in values among groups A and B.

### Outer Macular Thickness

The mean outer macular thickness (outer 6 mm) of the superior, inferior, temporal and nasal quadrants among groups A and B are expressed in the Table 3. There is statis tically signi ficant difference (p < 0.05) in values among groups A and B.

Both inner and outer macular thickness were lower among glaucoma patients (group A) than controls (group B) and the difference was significant (p < 0.05).

### Total Macular Volume (Table 4)

The mean total macular volume in POAG group was 6.18 ± 0.39 mm^3^ as compared to 6.6 ± 0.17 mm^3^ among the control eyes. The difference of 0.4 between the mean was statistically significant (p < 0.05).

**Table 1:** **Table 1:** Demographic characteristics of subjects

*Features*		*Group A*		*Group B*		*p-value*	
Age		50.48		50.52		0.962	
Male:Females patients		52:24		44:24		>0.05	
CD Ratio		0.63		0.38		<0.0001	
Mean IOP		23.22 mm Hg		14.45 mm Hg		<0.0001	

**Table 2:** **Table 2:** Comparison of inner macular thickness between the study groups

	*Groups*										
		*Group A*				*Group B*					
		*Mean*		*SD*		*Mean*		*SD*			
Temporal		233.09		24.47		256.23		9.6		<0.0001	
Superior		247.34		18.07		265.10		10.04		<0.0001	
Nasal		249.50		17.61		265.90		11.59		<0.0001	
Inferior		245.92		17.32		267.03		9.28		<0.0001	

**Table 3:** **Table 3:** Comparison of outer macular thickness between the study groups

	*Groups*										
		*Group A*				*Group B*					
		*Mean*		*SD*		*Mean*		*SD*			
Temporal		199.18		12.63		223.84		14.58		<0.0001	
Superior		216.85		13.99		235.52		13.18		<0.0001	
Nasal		231.38		17.79		259.90		37.04		<0.0001	
Inferior		203.06		23.79		230.68		14.32		<0.0001	

**Table 4:** **Table 4:** Distribution of total macular volume

		*Mean*		*Std. deviation*		*p-value*	
Group A		6.1814		0.39368		<0.0001	
Group B		6.6445		0.17213			

## DISCUSSION

Glaucomatous optic neuropathy results in death of RGC which are more densely populated in the macular region. On the basis of this anatomical relationship Zeimer et al. first observed the large losses in total macular thickness in patients with glaucomatous damage. There is a significant loss of RGCs in perifoveal region.^7^

Viviane Guedes^9^ et al in their cross-sectional OCT study of total 534 eyes of macular and RFNL thickness in normal and glaucomatous eyes concluded that both parameters showed statistically significant correlation with glaucoma. They concluded that macular thickness may be used as an additional parameter in clinical assessment of glaucoma. FN Kanadani^14^ et al in their study of glaucoma patients studied macular parameters using OCT and correlated the same with visual field changes and multifocal visual evoked potential (mfVEP ) of macular area. They found good correlation between assessment of structural changes of macula on OCT with functional changes on Visual field and mfVEP.

Sung MH et al and Delbarre M et al in their study evaluated the diagnostic use of macular thickness and RFNL thickness for diagnosing glaucoma. Both the studies found similar changes in macular thickness which correlated well with RFNL thickness among glaucomatous patients.^15,16^

Arvanitaki V et al^17^ used OCT scanning protocols in early glaucoma patients. The finding that retinal thickness was significantly lower in early manifest glaucoma patients and glaucoma suspects indicates that the transposition of the OCT fast RNFL thickness (3.4 mm) protocol from the peripapillary area to the perimacular area can be used for the early glaucoma diagnosis. Intraretinal changes in early glaucoma, likely precede nerve fiber changes. In our study there was difference in macular thickness in both outer and inner region which is similar with the studies published. The central macular thickness was not different between the glaucomatous and the control eyes and probably the reason is lack of ganglion cells in the central foveal region.

Our study showed that mean inner macular thickness among glaucoma patients was 243.96 m while among normal subjects was 263.56 m. The mean outer macular thickness among glaucoma patients was 212.61 m while among normal subjects was 237.48 m. Thus it was seen that macular thickness was decreased in glaucomatous patients as compared to normal subjects. These findings are in correlation with studies discussed above and published literature.^10,18^

Muscat S et al^19^ and Koozekanani D et al^20^ assessed the accuracy, precision, repeatability and reproducibility of OCT and found it as a useful tool in glaucoma. OCT macular and RFNL parameters may be useful in patients who may not be cooperative for visual field studies.

There are few studies which were specifically done to show correlation between macular volume and glaucoma status. Giovannini A et al showed in their study that the OCT macular volumes correlate significantly with glaucoma status.^21^ Lederer et al evaluated macular volume in normal, glaucoma suspect and glaucomatous subjects using a time domain OCT.^22^ Their results demonstrated a significant correlation between the macular volume and glaucoma status with decreased macular volume in patients with advanced disease as well as significant difference of macular volume between normal and glaucomatous eyes. In our study the macular volume in glaucomatous eye was 6.18 ± 0.39 m and in the control eyes 6.64 ± 0.17 m which was statistically significantly different as in studies published internationally.

The limitation of our study are that sample was small and time domain OCT was used. Nonavailability of SD-OCT was the reason for using Stratus OCT (Stratus OCT Model 300, Carl Zeiss, Meditec Inc. Dublin CA, USA). Future studies with large sample size may be required to validate our findings. Also correlation of structural OCT changes with functional parameters such as perimetry or multifocal visual evoked potential (mfVEP) may add to the strength of the study.

## CONCLUSION

We can state that macular thickness and volume shows a significant correlation with the glaucomatous damage. It may be useful method of documenting early glaucoma and monitoring progression. Thus, our study conclude that macular parameters, such as total macular volume, inner macu lar thickness and outer macular thickness can be used in addition to RNFL thickness to aid in the diagnosis of early glaucoma using OCT, in certain conditions, such as disk abnormalities or peripapillary atrophy, where RNFL parameters may be distorted macular parameters may be relied upon. However, these findings would require prospective long-term studies to see the progression of visual field defects or increase in glaucoma damage.
